# Case of Anthracycline-induced Cardiogenic Shock: A Call to Optimize Modifiable Cardiac Risk Factors Prior to Chemotherapy

**DOI:** 10.7759/cureus.4961

**Published:** 2019-06-20

**Authors:** Andre Gabriel, Bryan Stringer, Matthew J Hadfield, Mona Madady

**Affiliations:** 1 Internal Medicine, University of Connecticut Health Center, Farmington, USA; 2 Internal Medicine, St. George's University School of Medicine, St. George's, GRD

**Keywords:** anthracyclines, cardio-toxicity, hypertension, hyperlipidemia, chemotherapy, carvedilol

## Abstract

Anthracyclines, including doxorubicin, are an important class of chemotherapeutic agents. Their efficacy, however, is limited by cardiotoxicity. Risk factors for anthracycline-associated cardiotoxicity include dose, treatment-specific risk factors including adjunctive radiotherapy, patient-specific modifiable cardiac risk factors including hypertension, hyperlipidemia, diabetes mellitus, tobacco use and obesity, and patient-specific non-modifiable risk factors such as age. The reduction of treatment-specific factors is not always possible, but treatment and reduction of modifiable risk factors should always be an important aspect of the management plan and may reduce the risk of anthracycline-induced cardiotoxicity. We present the case of a 65-year-old male with multiple modifiable cardiovascular risk factors who developed cardiogenic shock shortly after the administration of combination therapy with anthracyclines for the treatment of Hodgkin’s lymphoma.

## Introduction

Anthracyclines are an important class of chemotherapeutic agents and a staple in many modern chemotherapy regimens. They are routinely used in the treatment of lymphomas and solid tumors. They exert their anti-cancer properties through a variety of mechanisms including the inhibition of DNA-topoisomerase II, the production of reactive oxygen species, and mitochondrial dysfunction [1]. The primary target of these agents are rapidly proliferating cells, as would be found in malignancy. Unfortunately, the deleterious effects are not limited to the cancer cells. Although anthracyclines’ primary mechanism of action is through DNA damage of targeted cancer cells, other cells such as cardiomyocytes are also negatively affected by free radical production and mitochondrial dysfunction [1]. Since cardiomyocytes have a high concentration of mitochondria, it is believed that they are highly sensitive to these agents and hence the increased frequency of cardiotoxicity [1].

The primary and greatest risk for doxorubicin cardiomyopathy is dose [2]. According to previous studies, incidence of cardiomyopathy is approximately 5% at 400 mg/m2 and increases to 48% at 700 mg/m2 [2]. Cardiotoxicity at doses less than 100 mg/m2 is exceedingly rare and carries an odds ratio of 1 [1]. Although acute toxicity does occur, the negative effects of anthracyclines are typically seen after many doses or the completion of treatment [1]. This can be explained by the cumulative dose effect. In the cases of acute toxicity, the role that additional risk factors play in increasing the risk of cardiotoxicity is unclear.

Patient-specific non-modifiable risk factors for cardiotoxicity from doxorubicin include age (greater than 65 or less than 18), having multiple cardiac risk factors and pre-existing cardiac dysfunction [3]. Treatment-specific factors include adjunctive radiotherapy, radiotherapy of the mediastinum, and combination therapy with other cardiotoxic agents such as trastuzumab [3]. Reduction of treatment-specific factors is not always possible based on cancer-specific factors. For example, hyperleukocytosis as seen in acute leukemia is associated with elevated mortality due to its ability to induce leukostasis, disseminated intravascular coagulopathy and tumor lysis syndrome and requires prompt induction chemotherapy [4]. Additionally, the use of anthracyclines in high-risk age groups and in persons with pre-existing cardiac dysfunction is often necessary and cannot be avoided. This often leaves cardiac risk factors as the only modifiable part of a treatment regimen.

Cancer patients often have concurrent cardiac risk factors including hyperlipidemia, hypertension, tobacco use, and obesity. For example, breast cancer and cardiovascular disease (CVD) have many overlapping risk factors [5]. The exact mechanism or the cumulative effect these risk factors have on exacerbating anthracycline toxicity is not known; however, treatment and reduction of these associated risk factors should be an important aspect of these patients’ management plan. In this case report, we examine the case of a 65-year-old male with multiple modifiable cardiovascular risk factors, who developed cardiogenic shock shortly after administration of combination therapy with anthracyclines for the treatment of Hodgkin’s lymphoma.

## Case presentation

**Figure 1:**
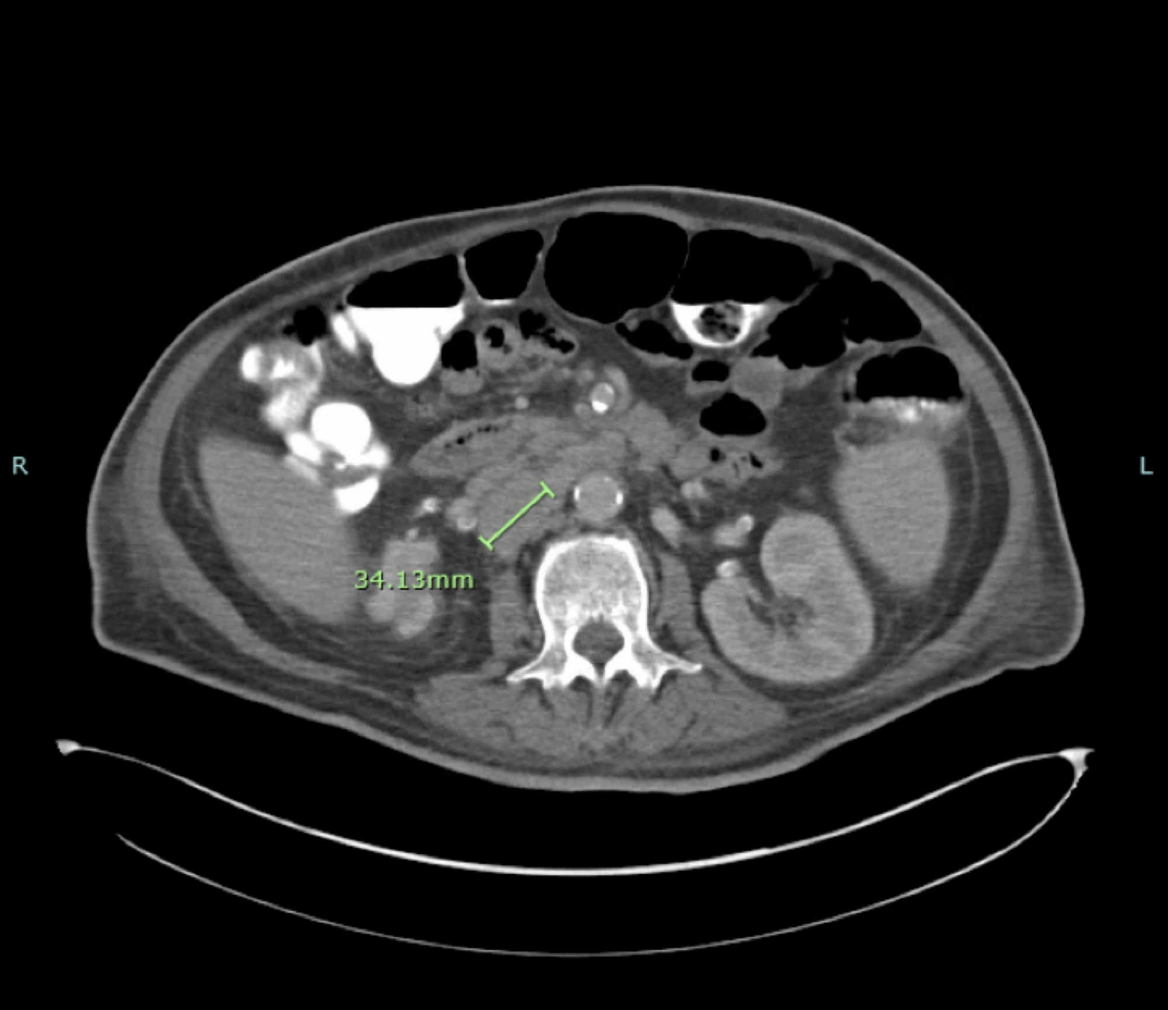
CT of the abdomen and pelvis demonstrating extensive retroperitoneal lymphadenopathy. Outlined in green is the largest node in the retrocaval area measuring 3.4 cm

A 65-year-old male with a past medical history of hypothyroidism, obesity, hyperlipidemia, hypertension, and type II diabetes presented to the emergency room with increasing weakness and decreased oral intake. He admitted to increasing lethargy, 40 lbs of unintentional weight loss, and had also been experiencing nausea and vomiting. At the time of admission, his vital signs were within normal limits with the exception of a low-grade temperature of 100.3F. His initial physical exam was significant for general pallor and abdominal distension, but was otherwise unremarkable. Laboratory work performed at that time showed pancytopenia with leukocytes of 3.4 g/dl, hemoglobin of 8.9 g/dl (baseline of 10.5 g/dl from three months prior), hematocrit of 27.8% and platelet count of 123 x 10*3/uL. He was also noted to have modest elevation in liver enzymes and lactate dehydrogenase level of 348 U/L. The patient underwent a computed tomography (CT) scan of the abdomen and pelvis, which revealed multiple enlarged pre-aortic, aorto-caval and retro-caval lymph nodes, with the largest node located in the retro-caval region, measuring 3.4 cm (Figure 1). The scan was also remarkable for moderate splenomegaly. Subsequent CT scan of the thorax revealed mediastinal and right hilar lymphadenopathy. Due to concern for underlying malignancy, the inpatient oncology team was consulted.

**Figure 2:**
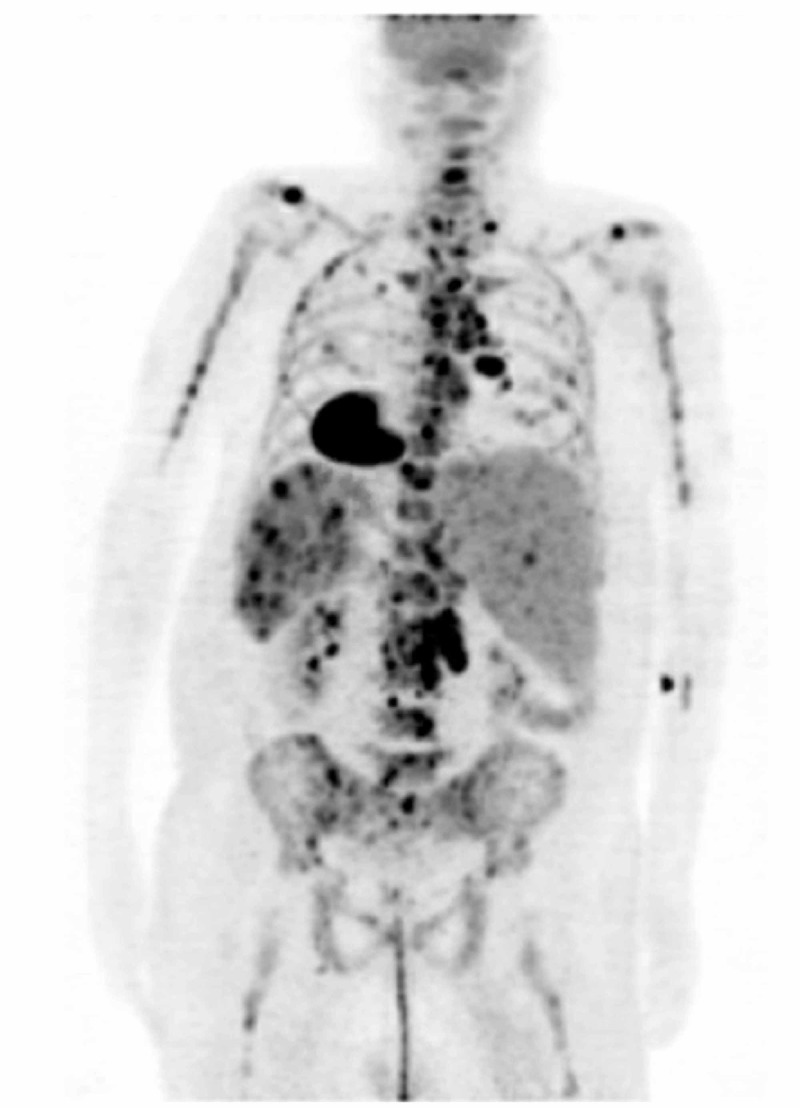
PET study demonstrating widespread involvement of bone, liver, spleen, right hilum, mediastinum and retroperitoneum PET - positron emission tomography

The patient underwent a positron emission tomography (PET) scan, which revealed widespread uptake including lesions in the bone, liver, spleen, as well as mediastinal and retroperitoneal lymph nodes with additional involvement of the right hilar lymph nodes (Figure 2). Tissue diagnosis was made after the patient underwent mediastinoscopy for lymph node sampling. Pathology was consistent with classic-type Hodgkin’s lymphoma, staged at IVB by Ann Arbor criteria. At this point, the patient’s condition started to deteriorate. He developed persistent fevers with hypotension and decreased urine output and required upgrade to ICU level of care for vasopressor support.

Once he was successfully weaned off vasopressor support and his overall condition appeared to improve, the patient was then initiated on adriamycin, bleomycin, vinblastine and dacarbazine (ABVD) for treatment of lymphoma. An echocardiogram was performed before the initiation of chemotherapy and demonstrated normal left ventricular ejection fraction estimated at 55%-65% (Video 1). There was trivial pericardial effusion; however, no other abnormalities were noted. Given his normal cardiac function the patient was deemed appropriate for chemotherapy treatment. Just prior to starting chemotherapy the patient had a precipitous drop in hemoglobin and hematocrit and overt melena that required blood transfusion before initiating chemotherapy.

**Video 1:** Initial echocardiogram demonstrating normal left ventricular systolic function with an estimated ejection fraction of 55%-65%

The patient received his first dose of ABVD (dose adjusted for elevated bilirubin) and tolerated the treatment without any issues. The day following the initiation of therapy, the patient was found to be obtunded, hypotensive, and tachycardic. He did not respond to fluid resuscitation and required intubation for worsening hypoxia and airway protection. A CT scan was performed due to concern for pulmonary embolism, which was subsequently found to be negative. The patient was profoundly fluid overloaded at this point and did not respond to aggressive diuresis with furosemide, metolazone or bumetanide. The patient was initiated on continuous venovenous hemofiltration (CVVH) with no improvement in overall condition. Due to profound hypotension, the patient required three vasopressors for hemodynamic support but remained hypotensive. An echocardiogram revealed severe decrease in left ventricular ejection fraction to 15%-25% with Simpson’s biplane ejection fraction of 26% (Video 2). The patient continued to decline and was unable to maintain oxygen saturations despite aggressive ventilator settings. The final cause of death was deemed to be cardiogenic shock secondary to receiving adriamycin in the setting of newly diagnosed IVB Hodgkin’s lymphoma.

**Video 2:** Echocardiogram after the initiation of chemotherapy demonstrating severe reduction in left ventricular function with estimated ejection fraction of 15%-25%

## Discussion

Our patient developed cardiogenic shock after receiving a single dose of ABVD with adriamycin for Hodgkin’s lymphoma and subsequently died as a result of dilated cardiomyopathy. Although he had an elevated risk profile that included advanced age, hypertension, hyperlipidemia, and type 2 diabetes, the occurrence of cardiotoxicity was unexpected.

As discussed previously, the greatest factor in anthracycline toxicity is the cumulative dose administered, with toxicity increasing in a stepwise manner. The deleterious effects of these agents typically manifest after treatment is completed and often become clinically significant only years after therapy [1]. Cases of acute onset of doxorubicin toxicity are extremely rare but have been reported in several case reports over the years. Hayek et al. described a case of acute doxorubicin-induced cardiotoxicity following the administration of 50 mg/m2 of doxorubicin while using hepatic artery chemoembolization treatment for carcinoid tumor [6]. Bristow et al. described a similar case of a patient suffering a myocardial infarction within 24 hours of receiving 60 mg/m2 of doxorubicin [7].

Given the established cardiotoxic effects of anthracyclines, strategies have been developed to reduce the risk of toxicity and to detect early signs of toxicity. Treatment-specific strategies include use of infusion therapy in place of bolus administration, use of liposomal encapsulation of anthracyclines, and the use of cardio-protective agents, such as dexrazoxane [8]. Additionally, the use of echocardiography with strain imaging and monitoring of cardiac biomarkers has allowed for the early detection of subclinical signs of myocardial injury [9]. Despite being effective, these strategies often do not take into consideration the treatment of modifiable cardiac risk factors such as hypertension. In a study by Szmit S et al. it was found that hypertension itself intensified the effects of doxorubicin toxicity and hence caused delay in treatment and subsequent worse outcomes [10].

In patients receiving chemotherapeutic agents, such as the patient presented here, we believe that pretreatment with and concurrent use of agents targeting pre-existing cardiac risk factors such as beta-blockers, ace inhibitors, and statins may play a significant role in decreasing risk of toxicity, although this was challenging and not feasible in our patient, given his overall clinical picture. Pretreatment with these agents as a means of decreasing cardiotoxicity from anthracyclines has shown to be promising in research, but remains underutilized in practice and not yet part of standard guideline therapy. Furthermore, how these agents would decrease the risk of acute doxorubicin toxicity is not known. A study by Kalay et al. showed that prophylactic beta-blocker use decreased the incidence of reduced ejection fraction [11]. Avila et al. also showed that prophylactic use of carvedilol was associated with lower incidence of early onset diastolic dysfunction and lower levels of cardiac biomarkers [12]. Additional studies also suggest that statin therapy decreases the incidence of heart failure in breast cancer patients treated with anthracyclines [13]. These studies were not robust, however, and were too small to formulate guidelines. Current randomized stage III trials by the National Cancer Institute (NCI) to investigate the efficacy of carvedilol in preventing cardiac toxicity in patients with metastatic Her-2 positive breast cancer are ongoing.

## Conclusions

The use of prophylactic agents targeting pre-existing cardiac risk factors is not currently part of guideline therapy when using anthracyclines, but given their potential benefits, this may soon change. In our patient, it was not possible to implement these preventative strategies given hypotension and impaired liver function. Also, given the rarity of acute doxorubicin toxicity at such low doses, the use of agents may not have been justified. Nonetheless, when able to safely implement these medications, providers should strongly consider their use to prevent cardiotoxicity.
